# Advances in preclinical and clinical studies of oncolytic virus combination therapy

**DOI:** 10.3389/fonc.2025.1545542

**Published:** 2025-02-07

**Authors:** Wenlong Du, Jintong Na, Liping Zhong, Pumin Zhang

**Affiliations:** ^1^ State Key Laboratory of Targeting Oncology, National Center for International Research of Bio-targeting Theranostics, Guangxi Key Laboratory of Bio-targeting Theranostics, Collaborative Innovation Center for Targeting Tumor Diagnosis and Therapy, Guangxi Talent Highland of Major New Drugs Innovation and Development, Guangxi Medical University, Nanning, China; ^2^ Pharmaceutical College, Guangxi Medical University, Nanning, Guangxi, China

**Keywords:** oncolytic virus, tumor therapy, combined treatment, clinical trials, immunity

## Abstract

Oncolytic viruses represent a distinct class of viruses that selectively infect and destroy tumor cells while sparing normal cells. Despite their potential, oncolytic viruses encounter several challenges as standalone therapies. Consequently, the combination of oncolytic viruses with other therapeutic modalities has emerged as a prominent research focus. This paper summarizes the tumor-killing mechanisms of oncolytic viruses, explores their integration with radiotherapy, chemotherapy, immune checkpoint inhibitors, CAR-T, and CAR-NK therapies, and provides an overview of related clinical trials. By synthesizing these advancements, this study seeks to offer valuable insights for the clinical translation of oncolytic virus combination therapies.

## 1 Introduction

Oncolytic viruses offer a novel and promising approach to cancer therapy. They selectively infect and destroy tumor cells, sparing normal cells in the process (1). As the number of oncolytic viruses approved by the Food and Drug Administration (FDA) for clinical use continues to grow, interest in this therapeutic strategy has markedly increased. Oncolytic viruses can be administered as monotherapy or combined with radiotherapy, chemotherapy, immunotherapy, or cell-based therapies, presenting promising prospects for cancer treatment. Currently, Several oncolytic viruses are employed, such as adenovirus (Ad) (2), herpes simplex virus (HSV) (3), vaccinia virus (VV) (4), reovirus (5), poliovirus (6), coxsackie virus (CV) (7), Newcastle disease virus (NDV) (8), vesicular stomatitis virus (VSV) (9), myxoma virus (10) and some Senteroviruses (11).

**Figure 1:**
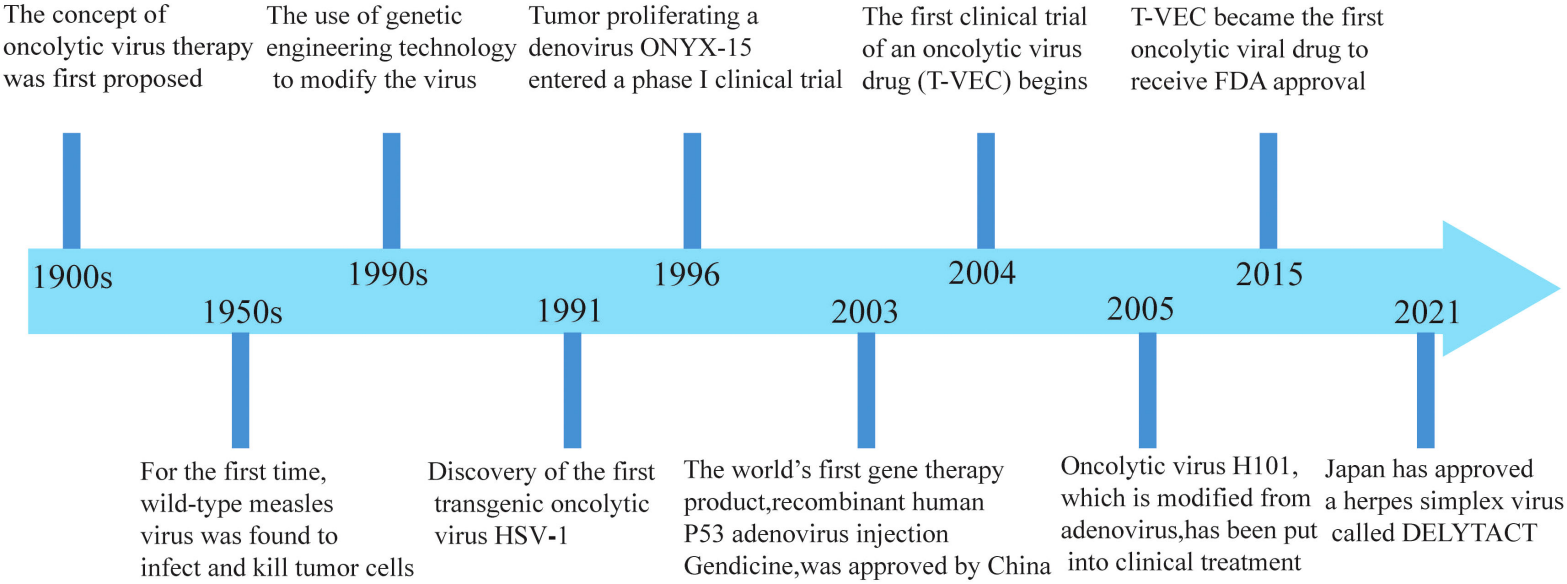
Significant milestones in the development of oncolytic viruses.

Research on oncolytic viruses began in the early twentieth century, revealing that certain wild-type or naturally attenuated viral strains could effectively treat cancer (12). Over recent decades, significant advancements had been achieved in cancer therapy with oncolytic viruses, and the milestones are summarized in **Figure 1**.

The antitumor effects of oncolytic viruses extend beyond receptor expression, potential mutations or transcriptional resistance. Furthermore, these viruses can stimulate non-autoantigen responses and amplify antitumor immune (13, 14).

Preclinical and clinical evidence demonstrates that oncolytic viruses can inhibit tumor growth through via multiple mechanisms. Nonetheless, several challenges limit their clinical translation. However, the clinical application of oncolytic viruses is hindered by challenges such as safety concerns, immune evasion, large-scale production, and clinical trials design (1, 15). Integrating oncolytic viruses with complementary therapies may enhance their efficacy and address existing challenges.

## 2 Factors influencing the anti-tumor effects of oncolytic viruses

### 2.1 Induction of tumor cell lysis

Oncolytic viruses proliferate rapidly within tumor cells competing for biomolecules and energy, ultimately leading to host cell damage. Oncolytic virus releases progeny viruses after lysis of tumor cells, which infect nearby tumor cells and gradually metastasize to distal tumor cells until cleared by the host immune system (16). Viral replication of whin tumor cells alters their metabolic profile, inhibiting DNA repair, disrupts cell cycle regulation, and promotes apoptosis.

**Figure 2:**
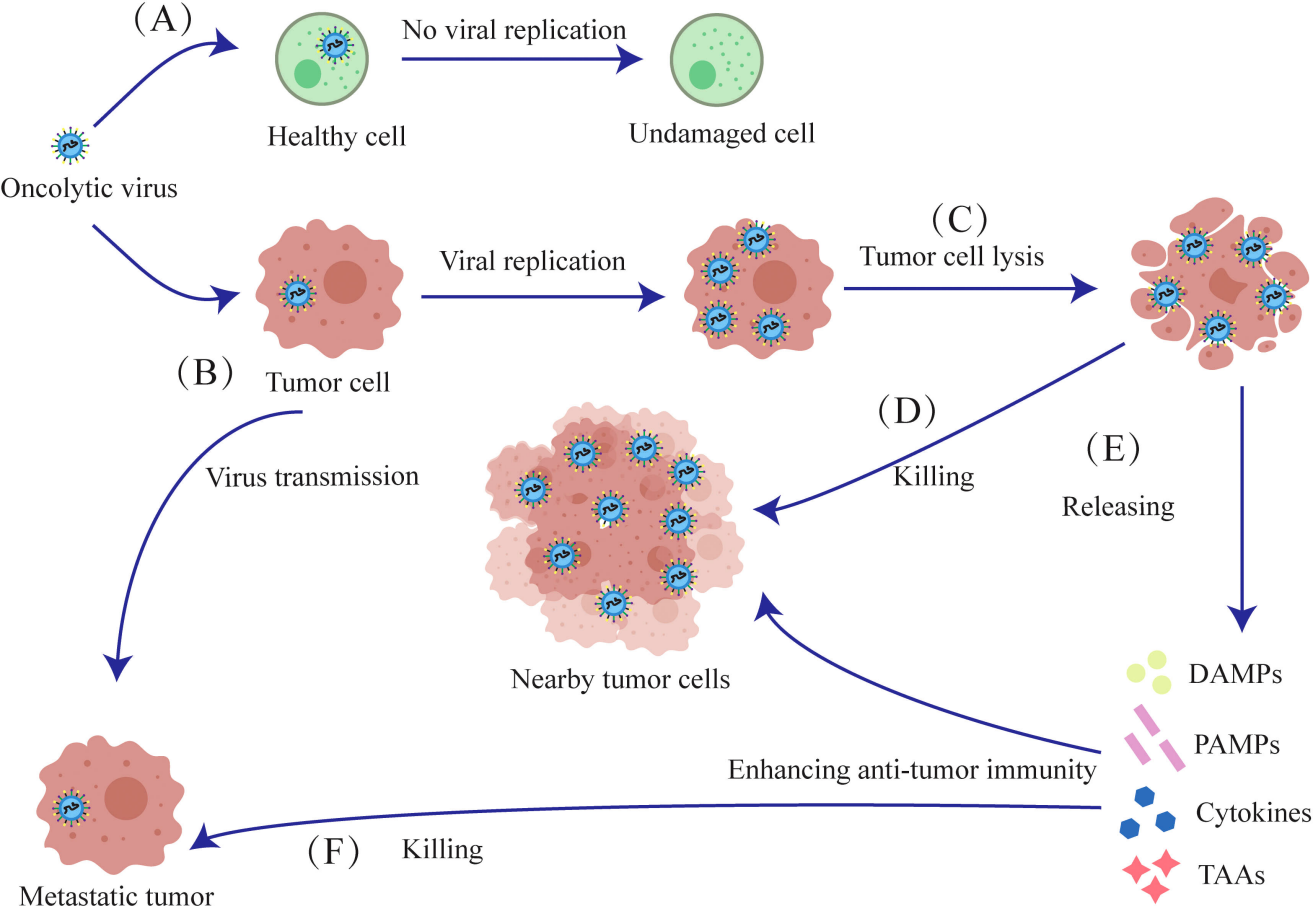
Mechanisms of oncolytic viruses. **(A)** The oncolytic virus has no destructive effect on normal cells. **(B)** The oncolytic virus selectively infects tumor cells, initiating viral replication within them. **(C)** Oncolytic viruses proliferate and lyse tumor cells. **(D)** The released oncolytic viruses then infect nearby tumor cells and lyse them. **(E)** Tumor cells lysed by oncolytic viruses release Cytokines, TAAs, DAMPs and PAMPs, which induce anti-tumor immunity against proximal and metastatic tumors. **(F)** After lysing tumor cells to release antigens and cytokines, oncolytic viruses further infect and kill metastatic tumors.

Finally, tumor cells destroyed by oncolytic viruses also release damage-associated molecular patterns (DAMPs) and pathogen-associated molecular patterns (PAMPs), which intensify the immune response against the surrounding tumor cells (**Figure 2**).

While oncolytic virus generally spare healthy cells, excessive oncolytic activity may result in off-target effects or heightened toxicity, potentially causing damage to normal tissues (17). The efficacy of oncolytic viruses is affected by factors such as virus type, injection dose, tumor cells sensitivity, and genetic modifications (18, 19).

### 2.2 Tumor immune response

Oncolytic viruses can elicit specific B-cell and T-cell responses against tumor antigens, and potentially prevent long-term tumor recurrence. B lymphocytes primarily secrete antibodies and eliminate tumor cells through antibody-mediated mechanisms. Cytotoxic T lymphocytes (CTLs) directly recognize and destroy tumor cells, while helper T cells regulate and amplify the immune response. Innate immune cells, such as macrophages, natural killer (NK) cells and dendritic cells (DCs) can also directly kill tumor cells and secrete cytokines that amplify adaptive immune responses or sensitize tumor cells to viruses (20, 21).

**Figure 3:**
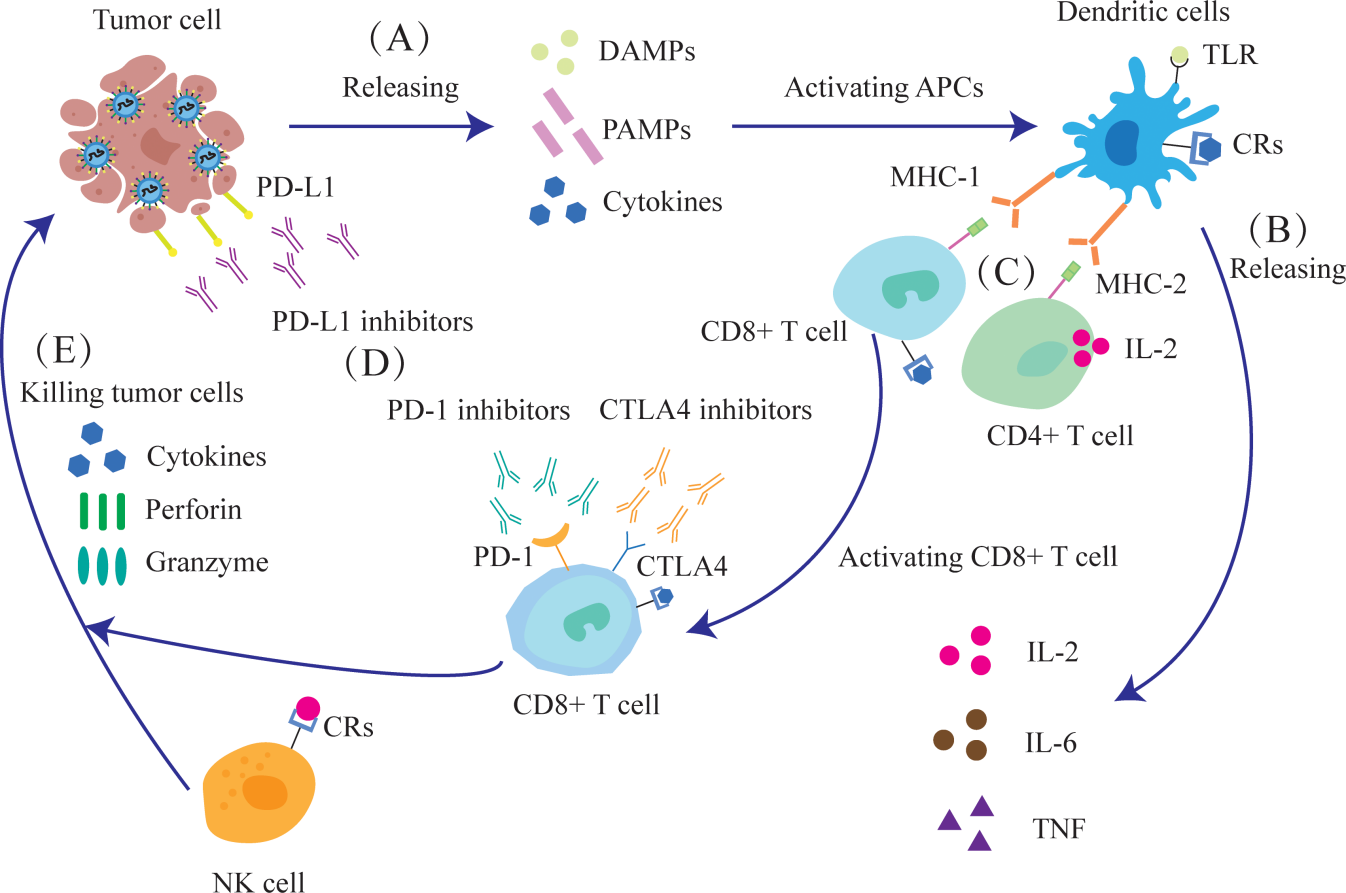
Oncolytic viruses can enhance the anti-tumor immune response and improve the effect of immunotherapy. **(A)** The destruction of tumor cells releases DAMPs, PAMPs and cytokines. **(B)** APCs, such as DCs, are activated and release IL-2, IL-6, and tumor necrosis factor (TNF). **(C)** After dendritic cells mature, they activate CD4+ and CD8+ T cells through MHC. **(D)** Oncolytic virus infection leads to increased expression of the immune checkpoint molecules PD-1, PD-L1 and CTLA-4, as well as enhanced sensitivity of tumor cells to immune checkpoint inhibitors (ICIs). **(E)** NK cells, together with activated CD8+T cells, can release cytokines, perforin and granzyme to kill tumor cells.

Oncolytic viruses induce the release of toll-like receptor (TLR) ligands, PAMPs and DAMPs from the infected tumor cells, which then activate antigen-presenting cells (APCs), NK cells and T cells. TLR ligands can counteract tumor-induced immunosuppression by modulating cytokines (22). Furthermore, DCs expressing the Major Histocompatibility Complex-1(MHC-1) and MHC-2 receptors respectively activate the CD8+ and CD4+T cells by presenting antigens. NK cells and activated CD8+T cells synergistically release perforin, granzyme and cytokines that directly kill tumor cells (**Figure 3**).

Oncolytic viruses elicit a host immune response that suppresses the tumor-killing effects, but this can be mitigated by combining oncolytic virus with low-dose chemotherapy or transforming growth factor beta (TGF-β) (23, 24). These agents can transiently suppress immune response, thus ensuring survival of the virus particles till they reach the tumor site. However, the precise safe dosage of these drugs still required further investigation.

While anti-viral immunity impairs efficacy, tumor-killing responses can be enhanced by immune stimulation. Tumor regression can be enhanced by increasing their therapeutic dose, immunostimulants immunostimulants as adjuvants (25, 26), or engineering recombinant viruses to express immunostimulatory genes or modified promoter elements (27). Interestingly, a virus that is oncogenic in one host may exert oncolytic properties against various tumors in another host (28).

The expression of cytotoxic proteins can also boost the efficacy of oncolytic viruses. For instance, adenovirus death protein (ADP) is a glycoprotein that effectively lyses cells and releases viral particles during advanced stages of infection. Consequently, viruses overexpressing ADP can spread more rapidly and efficiently within tumors (29).

In a Phase I clinical trial, patients with malignant pleural mesothelioma received intrathoracic oncolytic virus therapy, which reduced tumor cell density and increase the density of multiple immune cells (30).

### 2.3 Recombinant oncolytic virus

Recombinant oncolytic virus strains are develop by incorporating target genes or expression elements in the viral genome. These engineered viruses produce supplementary proteins in the host tumor cells, which enhance the anti-tumor efficacy of oncolytic viruses or confer additional therapeutic attributes (31).

The oncolytic vaccinia virus represents a promising recombinant strain. Studies have shown that vaccinia virus, when armed with IL-2, IL-15 or HBD2 is highly effective at recognizing and killing tumor cells (32–34). Notably, recombinant vaccinia virus strains expressing bacterial flagellin have demonstrated oncolytic effects in solid tumor models (35).

Newcastle disease virus (NDV) is the longest-used oncolytic virus in clinical trials and has a well-established safety record as a monotherapy, attributed to its robust induction of antiviral responses in non-transformed mammalian cells (36). NDV can also be recombined in many ways, for instance, an NDV strain expressing matrix metalloproteinase (MMP) 8 enhances viral accumulation in tumors, thereby improving oncolytic efficacy. Additionally, NDV strains have been engineered to promote the release of IFNγ from virus-infected melanomas cells (37, 38).

In addition, many oncolytic viruses have been successfully reprogrammed, and it is believed that more successful novel oncolytic viruses will be designed for preclinical and clinical trials in the future.

### 2.4 The tumor microenvironment

The tumor microenvironment (TME) is a complex network of tumor and stromal cells that fosters the growth and survival of cancer cells. Depending on cytokine profile and infiltration of immune cells, the TME can be categorized as immunologically “cold” or “hot”.

A cold TME is defined by elevated levels of inhibitory cytokines and immune checkpoint molecules, along with high infiltration of immunosuppressive cells, which hinder the immune system from accurately identifying and killing tumor cells (39). Conversely, a hot TME facilitates detection and elimination of tumor cells via immune effector cells, pro-inflammatory cytokines, and immunostimulatory molecules.

Since hot tumors respond better to immunotherapy, transforming the TME from “cold” to “hot” can increase the effectiveness of oncolytic viruses by enhancing immune recognition and destruction of tumor cells (40).

**Figure 4:**
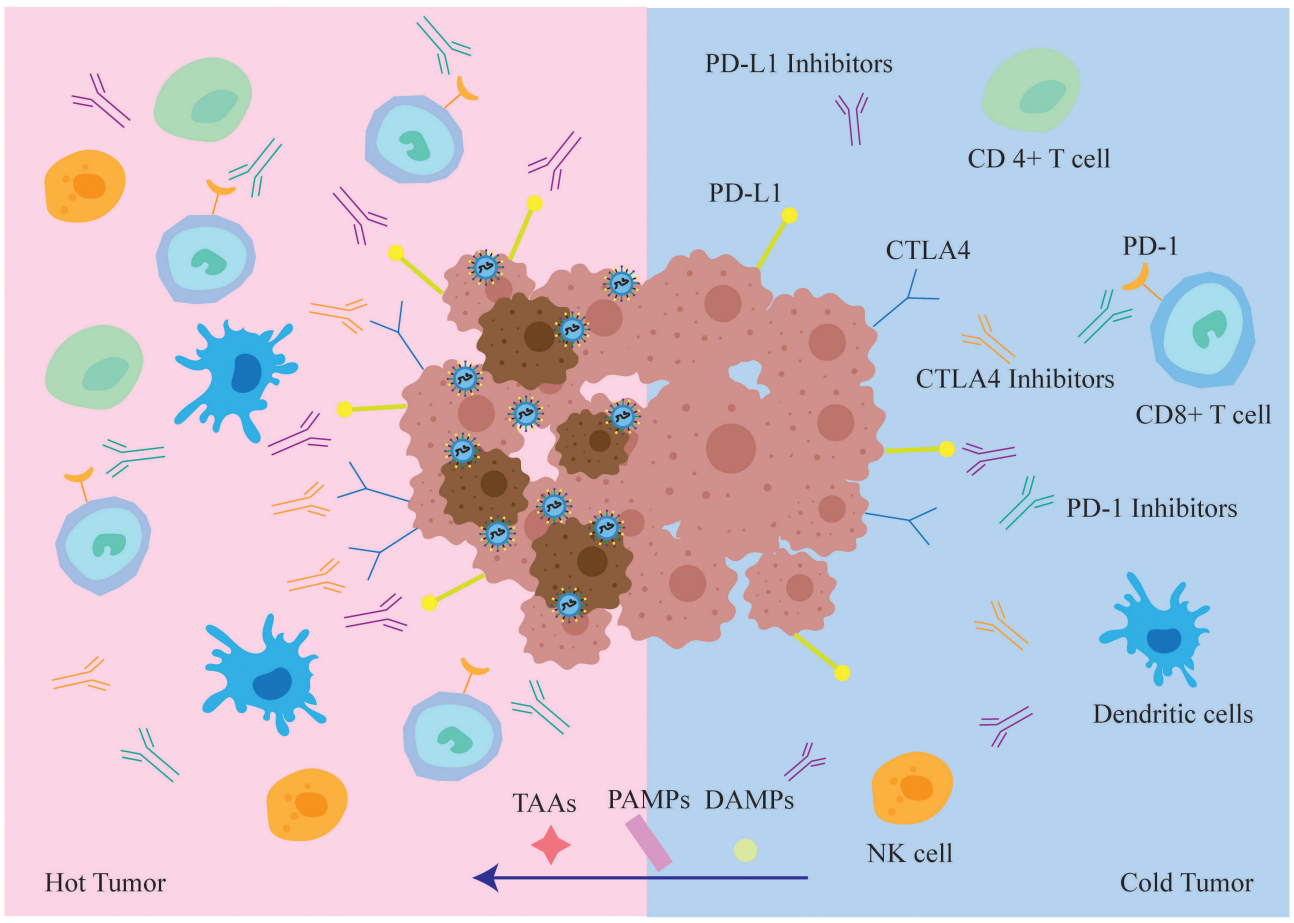
In immune “hot” TME, tumor cells infected with oncolytic virus are more easily recognized and cleared by the immune system. While oncolytic virus lyses tumor cells, it also draws various types of immune cells into the tumor microenvironment, transforming “cold” tumors into “hot” tumors and increasing the sensitivity of tumor cells to the immune checkpoint inhibitors ICIs. At the same time, TAA, PAMPs and DAMPs will also enter the “hot” tumor, TAA enhances the recognition and killing of tumor cells by the host immune system, whereas PAMPs and DAMPs enhance anti-tumor cellular immune responses.

Oncolytic viruses can transform the TME from “cold” to “hot”, and evoke an adaptive antitumor immune response by releasing tumor-associated antigens (TAAs), PAMPS, and DAMPS. These immunostimulatory molecules recruit antigen-presenting cells (APCs) to the tumor site (41), resulting in antitumor and antiviral responses (**Figure 4**). Thus, oncolytic viruses are a promising immunotherapeutic agent for tumor control.

Evidence indicates that viruses can transport genes encoding immunostimulatory molecules, such as cytokines, chemokines, and co-stimulatory receptors, to the TME, thereby enhancing antitumor immunity (42).

Viral particles and gene products released upon tumor cell lysis can also activate the immune system. Furthermore, oncolytic viruses can inhibit tumor angiogenesis, reduce tumor blood supply, and induce tumor hypoxia and nutrient deficiency by inactivating pro-angiogenic factors secreted by tumor cells. Virus-infected cells often trigger an inflammatory response that can lead to tissue damage. Oncolytic viruses can modulate the tumor microenvironment by regulating the production and release of inflammatory factors, reducing surrounding tissue damage, and thereby enhancing therapeutic efficacy.

Oncolytic adenoviruses engineered to locally express inflammatory cytokines IL-12 and PD-L1 blocking antibodies can repolarize TME, enhance CD8 T cell activity, and can kill tumor cells by altering the tumor microenvironment (43, 44).

## 3 Limitations of oncolytic virus monotherapy

Oncolytic viruses, as a novel anticancer approach, function by selectively infecting and lysing tumor cells. Despite their potential, the clinical efficacy of oncolytic virus monotherapy is significantly constrained by several factors:

### 3.1 Tumor heterogeneity

Oncolytic virus infection depends on specific viral receptors, the expression of which varies widely among tumor types and patients. This heterogeneity markedly impacts the efficacy of oncolytic viruses, complicating efforts to achieve consistent outcomes across tumors. Moreover, the high mutation rates of solid tumors frequently undermine the long-term effectiveness of oncolytic virus monotherapy (45).

### 3.2 Immune response

Infection of tumor cells by oncolytic viruses activates host immune responses, resulting in viral clearance and diminished tumor-killing efficacy. Tumors suppress immune responses via mechanisms like elevated PD-L1 expression or recruitment of regulatory T cells (Tregs), further reducing oncolytic virus efficacy (43, 46, 47).

### 3.3 Limitations in local and systemic efficacy

While effective at lysing local tumors, oncolytic viruses show limited efficacy in controlling metastatic tumors. Monotherapy frequently fails to comprehensively target metastatic lesions.

### 3.4 Balancing viral load and safety

Increasing the viral dose may improve therapeutic efficacy but also heightens the risk of adverse effects, including inflammatory responses, organ failure, and, in severe cases, death (17). Monotherapy struggles to balance the need for therapeutic efficacy with ensuring patient safety.

## 4 Combination of oncolytic virus therapy with other treatment approaches

Given the limitations of oncolytic virus monotherapy, combination therapy has become a pivotal approach in cancer treatment. Integrating multiple therapeutic approaches achieves synergistic effects, effectively killing tumor cells while addressing the limitations of monotherapy.

For example, radiotherapy and chemotherapy disrupt tumor barriers, enhancing viral penetration; immune checkpoint inhibitors modulate host immune responses, reducing the clearance of oncolytic viruses. Furthermore, the combination of oncolytic viruses with CAR-T or CAR-NK cells not only improves the tumor microenvironment but also releases viral particles and cytokines that further activate and enhance the functions of CAR-T and CAR-NK cells, ultimately improving the overall therapeutic efficacy.

### 4.1 Combination of oncolytic viruses with radiotherapy or chemotherapy

Although radiotherapy is routinely used for local tumors and early-stage disease, it has several disadvantages, including off-target effects on normal cells and the development of radio-resistance in the tumor cells, which compromise therapeutic outcomes. Furthermore, radiotherapy is largely ineffective against metastatic growth. Similarly, chemotherapeutic agents are typically non-selective, affecting normal tissues and resulting in systemic toxicity and side effects that can significantly impact patients’ quality of life. In addition, tumor cells frequently mutate, generating drug-resistant clones that diminish or nullify the therapeutic effects of chemotherapeutic drugs.

On the other hand, oncolytic viruses selectively infect and lyse tumor cells. Furthermore, while radiotherapy and chemotherapy are typically localized, oncolytic viruses can target metastatic tumors due to their ability to infect tumor cells. Thus, combining oncolytic viruses with chemotherapy or radiotherapy can provide both local and systemic disease control (48, 49).

Numerous preclinical studies have investigated the combination of oncolytic virus with radiotherapy and chemotherapy. Recent studies have shown that the combination of temozolomide or vincristine with oncolytic viruses can significantly kill mouse tumors (50, 51). At the same time, oncolytic viruses have been shown to enhance the efficacy of mitomycin and hydroxycamptothecin (52).

In clinical trials, H101, an oncolytic virus approved by China’s State Drug Administration, has been widely tested in combination with chemoradiotherapy. A number of clinical studies demonstrate the significant efficacy of oncolytic viruses in treating various tumors when combined with chemoradiotherapy (53–56).

Researchers have also combined a novel telomerase oncolytic virus with radiotherapy to treat patients with esophageal cancer, integrating recombinant technology with combination therapy (57).

Additionally, a prospective randomized phase 2 trial combining oncolytic viruses with radiotherapy reported a significant reduction in positivity in local biopsies (58). Reovirus has also demonstrated efficacy in clinical patients when combined with radiotherapy and chemotherapy (59, 60).

Numerous clinical studies on the combination of oncolytic viruses with chemoradiotherapy have reported favorable therapeutic outcomes with minimal side effects. Some clinical trials of oncolytic virus combined with chemoradiotherapy have been listed in **Table 1**.

**Table 1:** Clinical trials of oncolytic virus combined with radiotherapy and chemotherapy.

Oncolytic Virus	Cancers	Stage of Clinical Development	Therapy	Administration	Status	Trail No.
Ad (H101)	Genital Neoplasms	Not Applicable	radiotherapy	Intratumoral	Completed	NCT05051696
Ad (Enadenotucirev)	Locally Advanced Rectal Cancer	Phase I	radiotherapy	Intravenous	Completed	NCT03916510
Herpes simplex virus (GM-CSF)	Melanoma Stage IV	Phase I	radiotherapy	Intratumoral	Completed	NCT05068453
Ad (ADV/HSV-tk)	Metastatic Non-small Cell Lung Cancer/Metastatic Triple-negative Breast Cancer	Phase II	radiotherapy	Intratumoral	Completed	NCT03004183
Ad (NSC-CRAd-S-p7)	Glioma	Phase I	radiotherapy/chemotherapy	Intratumoral	Completed	NCT03072134
Vaccinia virus (GL-ONC1)	Cancer of Head and Neck	Phase I	radiotherapy/chemotherapy	Intravenous	Completed	NCT01584284
Ad(Ad-39yCD /mutTKSR1rep-ADP)	Non-small Cell Lung Cancer Stage I	Phase I	radiotherapy	Intratumoral	Completed	NCT03029871
HSV(G207)	Recurrent/progressive pediatric high-grade gliomas	Phase ll	radiotherapy	Intratumoral	Ongoing	NCT04482933
Ad(AIOCELYVIR)	Diffuse Intrinsic Pontine Glioma /Medulloblastoma	Phase I& II	radiotherapy	Intravenous	Ongoing	NCT04758533
Ad (LOAd703)	Pancreatic Cancer	Phase I& II	chemotherapy	Intratumoral	Ongoing	NCT02705196
Vaccinia virus(KM1)	Ovarian Cancer	Phase I	chemotherapy	Intraperitoneal	Ongoing	NCT05684731
Vaccinia virus(TG6002)	Glioblastoma/Brain Cancer	Phase I& II	chemotherapy	Intravenous	Completed	NCT03294486
Vaccinia virus(GL-ONC1)	Ovarian Cancer	Phase I& II	chemotherapy	Intraperitoneal	Completed	NCT02759588
Ad(LOAd703)	Pancreatic Adenocarcinoma /Ovarian Cancer/ Biliary Carcinoma /Colorectal Cancer	Phase I& II	chemotherapy	Intratumoral	Completed	NCT03225989
Ad(CG0070)	Non Muscle Invasive Bladder Cancer	Phase II& III	chemotherapy	Intratumoral	Completed	NCT01438112
HSV-2(OH2)	Melanoma	Phase III	chemotherapy	Intratumoral	Ongoing	NCT05868707
Measles virus (MV-NIS)	Ovarian/Fallopian/Peritoneal Cancer	Phase II	chemotherapy	Intraperitoneal	Ongoing	NCT02364713
HSV-1(HF10)	Pancreatic Cancer Stage	Phase I	chemotherapy	Intratumoral	Ongoing	NCT03252808
Ad(H101)	Intrahepatic Cholangiocarcinoma	Phase IV	chemotherapy	Intratumoral	Ongoing	NCT05124002
Vaccinia virus(GL-ONC1)	Ovarian Cancer	Phase III	chemotherapy	Intraperitoneal	Ongoing	NCT05281471
Ad(enadenotucire)	Locally Advanced Rectal Cancer	Phase I	radiotherapy/chemotherapy	Intravenous	Completed	NCT03916510
Herpes simplex 1 virus (Talimogene laherparepve)	Triple Negative Breast Cancer	Phase I& II	chemotherapy	Intratumoral	Completed	NCT02779855
Vaccinia virus (Pexa-Vec)	Hepatocellular Carcinoma	Phase III	chemotherapy	Intratumoral	Completed	NCT02562755
Reovirus(REOLYSIN)	Metastatic Colorectal Cancer	Phase I	chemotherapy	Intravenous	Completed	NCT01274624

### 4.2 Combination of oncolytic viruses and immune checkpoint inhibitors

The expression of immune checkpoint molecules on immune cells will inhibit their function preventing the body from generating an effective antitumor immune response. These checkpoints can be exploited by tumors to evade immune surveillance. can be exploited by tumors to evade immune surveillance. Immune checkpoint inhibitors (ICIs), also referred to as immune system modulators, target immune checkpoints to enhance the immune response or to relieve immune suppression. Commonly used ICIs include Nivolumab, Ipilimumab, Pembrolizumab and Atezolizumab.

ICIs re-engage T cell anti-tumor activity of T cells by reversing the immunosuppressive tumor microenvironment. Oncolytic viruses can stimulate immune response and promote immune cell infiltration, while ICIs amplify this effect by reducing inhibitory signals, thereby enhancing immune response and therapeutic effect. The response to ICIs depends heavily on the TME, where “hot” tumors respond better to treatment, whereas “cold” tumors are less responsive. Therefore, improving TME is a key strategy for enhancing treatment efficacy (61).

At present, the combination of oncolytic viruses and ICIs has demonstrated significant anti-tumor effects in preclinical studies across various tumor types. Recent studies have further validated this approach (62–64).

Numerous clinical trials have been conducted on combination therapies. At present, a variety of ICIs combined with oncolytic viruses are currently being evaluated in clinical trials.

Nivolumab, a PD-1 inhibitor, was the first approved immunotherapy drug in China. Nivolumab has been combined with various recombinant oncolytic viruses in multiple tumor types, demonstrating sufficient safety and significant tumor regression (65–67).

Ipilimumab, a monoclonal antibody targeting CTLA-4, enhances the immune system’s ability to kill cancer cells by inhibiting an immunosuppressive checkpoint. HF10 is a biologically selected replicating oncolytic virus derived from herpes simplex virus type 1 (HSV-1). So far, numerous clinical trials have evaluated the combination of HF10 and Ipilimumab demonstrating remarkable efficacy (68–70).

Pembrolizumab is the only PD-1 inhibitor globally and in China that has received first-line three indications and single-agent indications for advanced non-small cell lung cancer (NSCLC). Numerous clinical trials combining Pembrolizumab with oncolytic viruses have shown sustained responses, with clinical benefits observed even in refractory patients (71–73).

Atezolizumab, a PD-L1 inhibitor, was approved by the US FDA in 2016. Atezolizumab binds to PD-L1 on tumor cells and block its interaction with PD-1 on T cells and antigen presenting cells, thereby relieving immunosuppression and enhancing T cell-mediated tumor cell destruction. At present, clinical studies have proved that Atezolizumab combined with oncolytic virus is very effective and safe (74, 75).

Of course, in addition to the above major classes of immune checkpoint inhibitors, other ICIs such as the PD-1 antibody Camrelizumab and the PD-L1 antibodies Durvalumab and Avelumab also show promising research prospects, with several ongoing preclinical and clinical trials. The results of the experiment are also being expected.

Therefore, combining oncolytic virus with ICIs may enhance anti-tumor immune responses. Some clinical trials of oncolytic viruses combined with immune checkpoint inhibitors are listed in **Table 2**.

**Table 2:** Clinical trials of oncolytic viruses combined with immune checkpoint inhibitors.

Oncolytic Virus	Cancers	Stage of Clinical Development	Combination Drug Effect	Administration	Status	Trail No.
RT-01	Advanced Solid Tumor	Phase I	Nivolumab& ANTI-PD-1	Intravenous/Intravenous& Intratumoral	Completed	NCT05228119
RT-01	Advanced Solid Tumor	Phase I	Nivolumab& ANTI-PD-1	Intravenous/Intravenous& Intratumoral	Completed	NCT05122572
HSV-1(RP3)	Advanced Solid Tumor	Phase 1	Nivolumab& ANTI-PD-1	Intratumoral	Completed	NCT04735978
HSV-1(RP1)	Solid tumors	Phase I& II	Nivolumab& ANTI-PD-1	Intratumoral	Completed	NCT03767348
HSV-1 (Talimogene laherparepvec)	Breast Cancer	Phase I	Nivolumab& ANTI-PD-1 Ipilimumab& ANTI-CTLA4	Intratumoral	Completed	NCT04185311
HSV-1(RP3)	Squamous Cell Carcinoma of Head and Neck	Phase II	Nivolumab& ANTI-PD-1	Intratumoral	Ongoing	NCT05743270
Vaccinia virus (Pexa-Vec)	Metastatic Tumor/Advanced Tumor	Phase I	Ipilimumab& ANTI-CTLA4	Intratumoral	Completed	NCT02977156
Coxsackie virus (A21)	Uveal Melanoma	Phase I	Ipilimumab& ANTI-CTLA4	Intravenous	Completed	NCT03408587
HSV-1(HF10)	Malignant Melanoma	Phase II	Ipilimumab& ANTI-CTLA4	Intratumoral	Completed	NCT02272855
HSV-1(HF10)	Melanoma Stage III/IV	Phase II	Ipilimumab& ANTI-CTLA4	Intratumoral	Completed	NCT03153085
Vesicular stomatitis virus (VSV-hIFNβ-NIS)	B-Cell Non-Hodgkin Lymphoma/Histiocytic and Dendritic Cell Neoplasm/Myelodysplastic Syndrome/Previously Treated	Phase I	Nivolumab& ANTI-PD-1 Ipilimumab& ANTI-CTLA4	Intravenous	Ongoing	NCT03017820
HSV-2(OH2)	Melanoma	Phase I& II	Pembrolizumab& ANTI-PD-1	Intratumoral	Completed	NCT04386967
Ad(DNX-2401)	Glioblastoma/Gliosarcoma	Phase II	Pembrolizumab& ANTI-PD-1	Intratumoral	Completed	NCT02798406
APS9801	Advanced Metastatic Solid Tumors	Phase I	Pembrolizumab& ANTI-PD-1	Intratumoral	Completed	NCT03954067
Chimeric orthopox virus (CF33-hNIS)	Metastatic/Advanced Solid Tumors	Phase I	Pembrolizumab& ANTI-PD-1	Intravenous	Ongoing	NCT05346484
Ad (TILT-123)	Ovarian Cancer	Phase I	Pembrolizumab& ANTI-PD-1	Intratumoral/Intraperitoneal	Ongoing	NCT05271318
Vaccinia virus(TBio-1)	Solid Tumor	Phase I& II	Pembrolizumab& ANTI-PD-1	Intratumoral/Intravenous	Completed	NCT04301011
Coxsackie virus(CAVATAK)	Non-Small Cell Lung Cancer	Phase I	Pembrolizumab& ANTI-PD-1	Intravenous	Completed	NCT02824965
Vaccinia virus(BT-001)	Metastatic/Advanced Solid Tumors	Phase I& II	Pembrolizumab& ANTI-PD-1	Intratumoral	Ongoing	NCT04725331
Vesicular stomatitis virus(VSV-IFNβ-NIS)	Metastatic/Advanced Solid Tumors	Phase I& II	Pembrolizumab& ANTI-PD-1	Intravenous	Completed	NCT03647163
HSV(M032)	Glioblastoma Multiforme	Phase I& II	Pembrolizumab& ANTI-PD-1	Intratumoral	Ongoing	NCT05084430
MG1-MAGEA3	Metastatic Melanoma/Squamous Cell Skin Carcinoma	Phase III	Pembrolizumab& ANTI-PD-1	Intravenous	Completed	NCT03773744
Reovirus(REOLYSIN)	Pancreatic Adenocarcinoma	Phase I	Pembrolizumab&ANTI-PD-1	Intravenous	Completed	NCT02620423
MG1-MAGEA3	Non-Small Cell Lung Cancer	Phase I& II	Pembrolizumab& ANTI-PD-1	Intravenous	Completed	NCT02879760
Ad (LOAd703)	Pancreatic Cancer	Phase I& II	Atezolizumab& ANTI-PD-L1	Intratumoral	Ongoing	NCT02705196
HSV-1 (RP2, RP3)	Metastatic Colorectal Cancer	Phase II	Atezolizumab& ANTI-PD-L1	Intratumoral	Ongoing	NCT05733611
Ad (LOAd703)	Malignant Melanoma	Phase I& II	Atezolizumab& ANTI-PD-L1	Intratumoral	Completed	NCT04123470
HSV-1 (RP3)	Hepatocellular Carcinoma	Phase II	Atezolizumab& ANTI-PD-L1	Intratumoral	Ongoing	NCT05733598
Ad (TILT-123)	Melanoma/Head and Neck Squamous Cell Carcinoma	Phase I	Avelumab& ANTI-PD-L1	Intratumoral	Ongoing	NCT05222932
Reovirus (PeLareorEp)	Breast Cancer Metastatic	Phase II	Avelumab& ANTI-PD-L1	Intravenous	Completed	NCT04215146
Ad (H101)	Recurrent Cervical Cancer	Phase II	Camrelizumab& ANTI-PD-1	Intratumoral	Ongoing	NCT05234905
Ad (H101)	Bladder Cancer	Phase II	Camrelizumab& ANTI-PD-1	Intravesical	Ongoing	NCT05564897
M1-c6v1	Advanced/Metastatic Hepatocellular Carcinoma	Phase I	SHR-1210	Intravenous	Completed	NCT04665362
MEDI5395	Advanced Solid Tumors	Phase I	Durvalumab& ANTI-PD-L1	Intravenous	Completed	NCT03889275
Vaccinia virus (Pexa-Vec)	Refractory Colorectal Cancer	Phase I& II	Durvalumab& ANTI-PD-L1 Tremelimumab&ANTI-CTLA4	Intravenous	Completed	NCT03206073

### 4.3 Combination of oncolytic viruses with CAR-T cells

T cells are genetically engineered to express a chimeric antigen receptor (CAR) transgene and become chimeric antigen receptor T cells (CAR-T cells). CAR proteins are comprised of three components: the extracellular antigen-recognition domain of the single-chain fragment variable region (scFv), a transmembrane domain, and the intracellular CD3ζ domain (76). The design of CAR-T cells is complex and has evolved through five generations.

**Figure 5:**
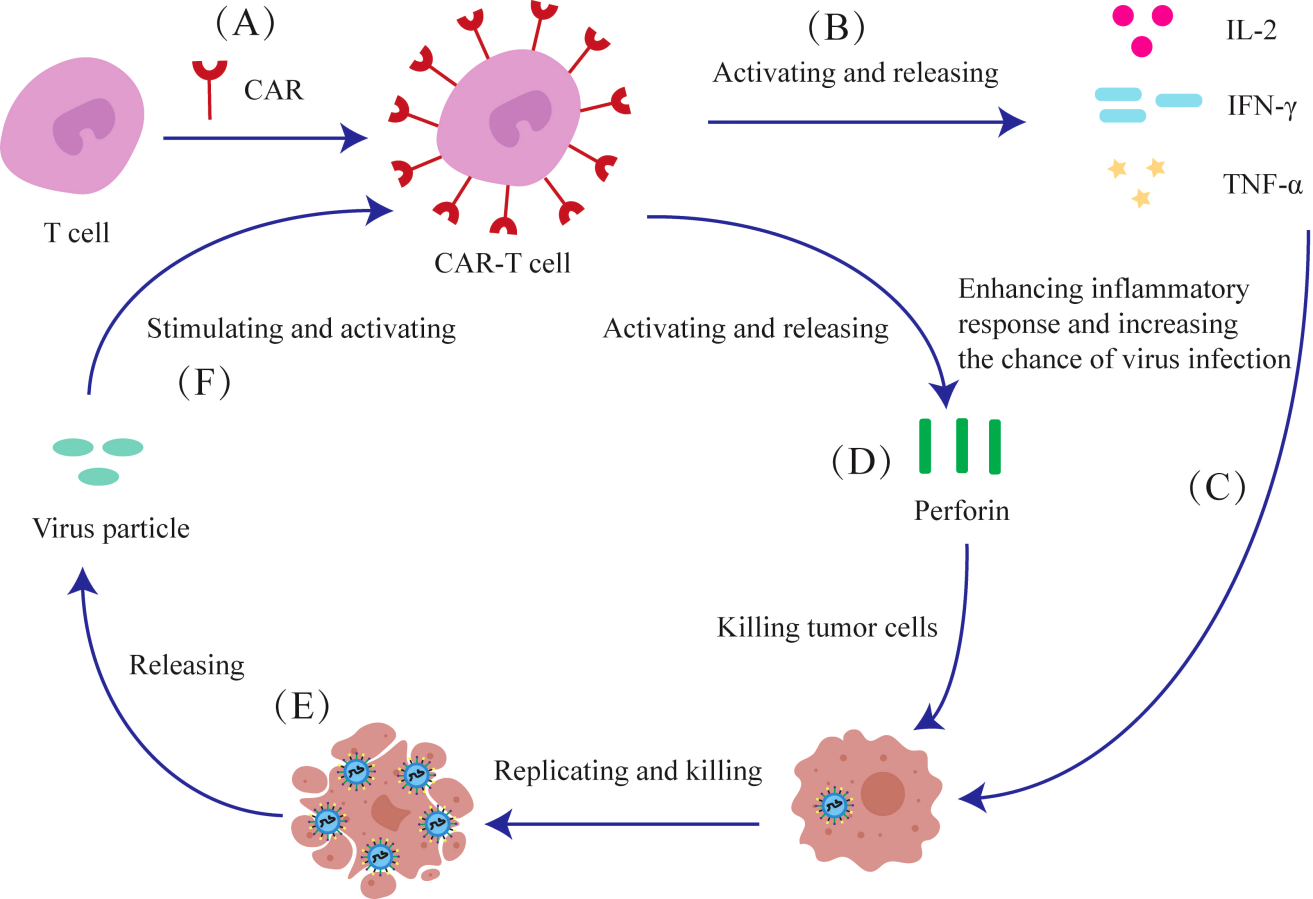
The anti-tumor mechanism of oncolytic viruses and CAR-T cells. **(A)** T cells are genetically modified to express CAR transgenes. **(B)** CAR-T cells can release IL-2, IFN-γ, and TNF-α after activation. **(C)** These molecules can intensify the inflammatory response in tumor cells, thereby increasing their susceptibility to viral infection. **(D)** CAR-T cells can release perforin to kill tumor cells. **(E)** Oncolytic viruses can release viral particles after they replicate and lyse tumor cells. **(F)** These viral particles can stimulate and activate CAR-T cells to produce effects.

Oncolytic viruses activate CAR-T cells and also guide them to the infected tumor cells. In turn, the activated CAR-T cells release cytokines, such as IL-2, IFN-γ and TNF-α, which enhance replication and infectivity of oncolytic viruses, thereby amplifying the overall therapeutic effect (**Figure 5**).

CAR-T cell therapy is a new cellular immunotherapy technique that integrates synthetic receptors into T cells, enabling them to recognize and kill tumor cells using homologous targeting ligands (77, 78). However, it was soon discovered that CAR-T therapy has limitations and side effects such as systemic toxicity and neurotoxicity, and the therapeutic effect may not be durable. At the same time, the complex construction process of CAR-T cells presents significant challenges for their application (79, 80).

Experimental data demonstrate the substantial clinical efficacy of CAR-T cells in treating hematologic malignancies (81). However, significant challenges remain in using CAR-T cells to treat advanced solid tumors. These challenges are primarily due to the low transport efficiency of CAR-T cells to TME, issues with antigen recognition, and structural differences between the structure of other solid tumors and hematologic tumors. Single therapy with CAR-T cells may not overcome these problems smoothly (82). However, with the FDA approval of the first oncolytic virus, T-VEC,the combination of oncolytic virus and CAR-T cells has officially opened the prelude. Recombinant oncolytic viruses may suppress local immunity, resulting in better therapeutic efficacy and persistence of CAR-T cells (83).

Oncolytic virus serve as reliable adjuvant for CAR-T cell therapy in solid tumors (84). Preclinical experiments have shown that oncolytic viruses loaded with IL-7 and CXCL11, in combination with CAR-T cells, significantly enhance tumor cell killing (85, 86).

At the same time, an interesting study in which oncolytic viruses expressing PD-L1 blocking micro-antibodies with CAR-T cells successfully controlled solid tumor growth (87). Another study combined CAR-T cells with oncolytic viruses equipped with the chemokine RANTES and the cytokine IL15, which improved the survival rate of tumor-bearing mice (88). Additionally, innovative strategies have been developed to encapsulate oncolytic viruses within CAR-T cells for treating solid tumors, yielding promising results (89).

Current studies suggest that the combination of oncolytic virus and CAR-T cells enhances therapeutic efficacy by enabling the virus to replicate and destroy tumor cells, stimulate immunity, and modulate the tumor’s immunosuppressive microenvironment, thus promoting CAR-T cell survival and activity.

Despite encouraging preclinical results, only one clinical trial investigating the combination of CAR-T cells and oncolytic viruses is ongoing, and further clinical trials are expected to follow. This may be due to the immunosuppressive tumor microenvironment, T cell depletion, or the absence of suitable antigen targets.

The summary of clinical trials of oncolytic virus combined with CAR-T cells for cancer treatment is shown in **Table 3**.

**Table 3:** Clinical trials of oncolytic viruses combined with CAR-T cells.

Oncolytic Virus	Cancers	Stage of Clinical Development	Patient type	Administration	Status	Trail No.
Ad(CAdVEC)	Solid Tumors	Phase I	HER2-positive	Intratumoral	Ongoing	NCT03740256

The slow progress of clinical trials combining CAR-T cells and oncolytic virus may be attributed to the systemic toxicity and neurotoxicity caused by CAR-T cells, which compromise their safety. In recent years, numerous patient deaths in CAR-T cells clinical trials have led to the emergency suspension of the experiment by FDA. In this way, attention has shifted to the carrier function of CAR T cells, using them to deliver oncolytic viruses to tumors to produce the effect of killing tumor cells. Studies have shown that CAR-T cells can deliver low doses of oncolytic viruses without affecting T cells quantity or function. CAR-T cells can be used as a carrier to release the virus to multiple tumor targets, thus further enhancing the role of killing tumor cells (90).

### 4.4 Combination of oncolytic viruses with CAR-NK cells

**Figure 6:**
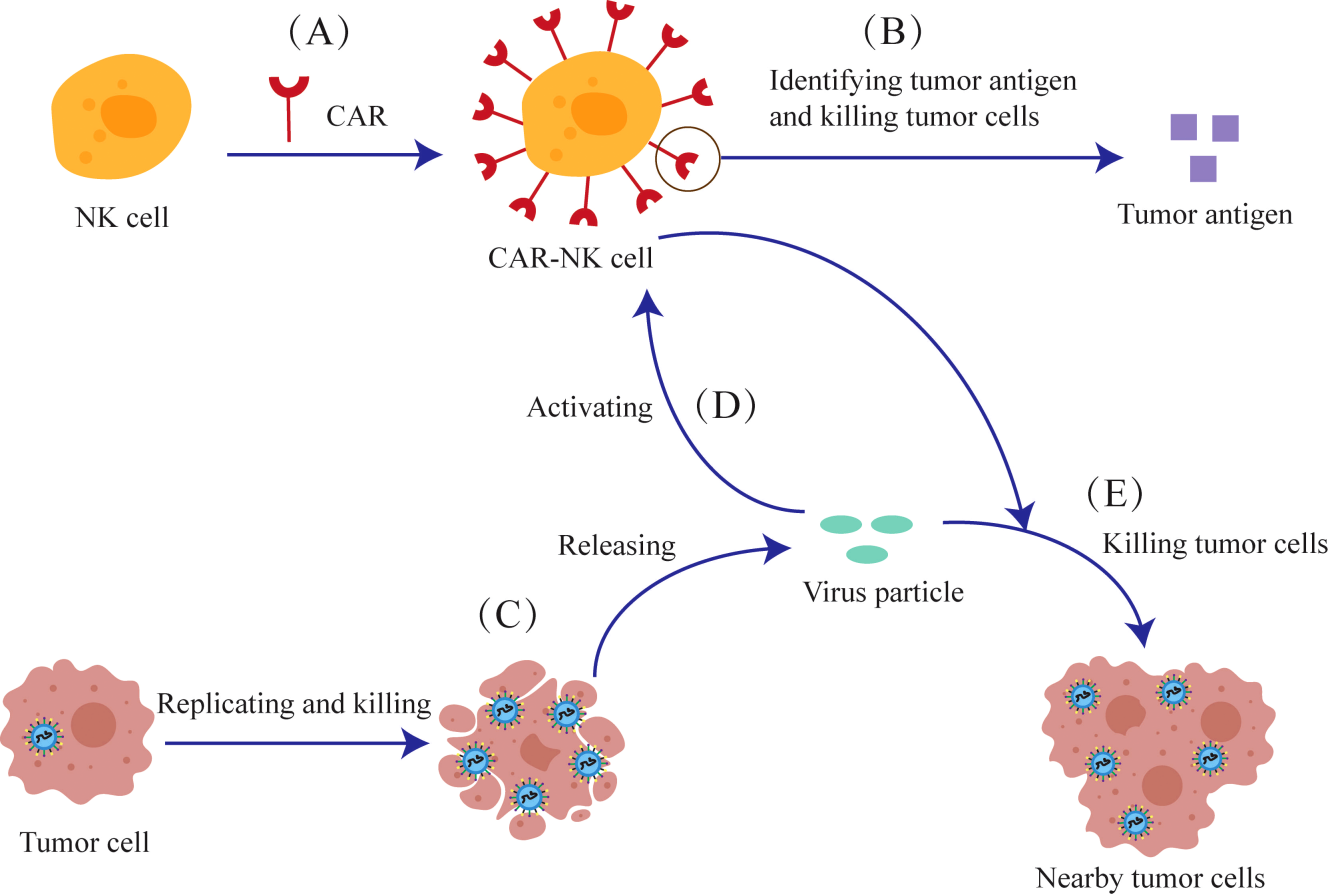
The anti-tumor mechanism of the combination of oncolytic viruses and CAR-NK cells. **(A)** NK cells are genetically modified to express CAR-Transgenes. **(B)** The CAR expressed on CAR-NK cells can recognize tumor antigens and kill tumor cells. **(C)** Oncolytic viruses can release viral particles after they replicate and lyse tumor cells **(D)** Viral particles can also activate CAR-NK cells **(E)** CAR-NK cells can kill nearby tumor cells together with viral particles.

Due to concerns about the safety of CAR-T cells, attention has shifted to NK cells that express a low level of PD-1, which promote the migration of dendritic cells and cause less immunosuppression. Genetically modified NK cells express CAR transgenes are termed Chimeric Antigen Receptor NK cells (CAR-NK cells).CAR-NK cells are engineered to recognize and annihilate cancer cells (**Figure 6**). The viral particles released from the lysed tumor cells can stimulate CAR-NK cells in a manner similar to the CAR-T cells.

CAR-NK cells are easier to construct than CAR-T cells and avoid issues such as alloreactivity, cytokine release syndrome, and graft-versus-host reactions. As long as NK cells are used, CAR-NK can be mass-produced. CAR-NK therapy is also significantly safer and does not induce strong neurotoxicity. At the same time, the survival cycle of NK cells is much shorter than that of T cells, and any off-target effects are rapidly cleared (91, 92).

As mentioned above, the shorter lifespan of NK cells necessitates multiple drug injections for CAR-NK therapy. But once the drug is injected multiple times, it will inevitably reduce safety. The continuous stimulation of immunity by oncolytic viruses helps extend the duration of CAR-NK cells without repeatedly injecting drugs.

At present, relevant preclinical studies have confirmed this view. EGFR-CAR constructs have been designed and transduced into NK cells to generate CAR-NK cells. The combination of second-generation EGFR-CAR NK cells and oncolytic herpes simplex virus to in treating breast cancer brain metastases in mouse models demonstrated significant tumor cell killing and improved survival (93). Another study showed that oncolytic viruses expressing IL15/IL15Rα, when combined with EGFR-CAR NK cells, induced a strong anti-tumor response in glioblastoma treatment (94).

In recent years, preclinical trials combining CAR-NK cells and oncolytic viruses have shown encouraging results. There are more and more clinical trials on CAR-NK cells, but the safety and efficacy of CAR-NK cells for clinical use remain subjects of debate. At present, no clinical trials have been conducted combining oncolytic viruses with CAR-NK cells.

## 5 Conclusions

In recent years, oncolytic viruses have received widespread attention as a promising cancer therapy. Meanwhile, genetic modification of oncolytic viruses can substantially enhance their anti-tumor efficacy.

This article reviews the factors influencing the anti-tumor efficacy of oncolytic viruses and their application in combination with other therapies, while also listing ongoing or completed clinical trials. The results showed that the combination of oncolytic viruses with other treatments was more effective than monotherapy. In the future, the design of more oncolytic viruses and their use in combination with other therapeutic approaches are expected to further enhance clinical efficacy and safety. In the future, We expect that oncolytic viruses will increasingly be combined with CAR-T cells and CAR-NK cells in clinical trials.

For the genetically modified part of the oncolytic viruses, utilizing better-targeted nanomaterials as a carrier to deliver the virus may enhance its efficacy, representing an innovative and more effective treatment strategy when combined with nanomaterials. At present, a team has started similar experiments, but further studies are needed to evaluate their effects (95).
